# Risk factors and long-term impact of anastomotic leak after colorectal cancer surgery in the elderly: a 5-year follow-up study

**DOI:** 10.1007/s10151-026-03397-3

**Published:** 2026-07-31

**Authors:** R. Blanco-Colino, G. Pellino, F. Marinello, M. Martí Gallostra, E. Mena Muñoz, M. Kraft, N. Ortega-Torrecilla, J. L. Sánchez-García, A. Solís Peña, F. Vallribera, E. Espín-Basany

**Affiliations:** 1 Department of Surgery, Universitat Autònoma de Barcelona, Barcelona, Spain; 2 Hepatobiliary and Transplant Surgery Unit, Royal Infirmary of Edinburgh, Edinburgh, UK; 3 Colorectal Surgery, Department of General and Digestive Surgery, Vall d’Hebron University Hospital, Barcelona, Spain

**Keywords:** Anastomotic leak, Colorectal surgery, Colorectal cancer, Elderly, Age, Frailty, Morbidity, Mortality

## Abstract

**Background:**

The elderly population represents an increasing proportion of patients undergoing surgery for colorectal cancer. Their risks and outcomes may differ from the general population. This study aimed to identify risk factors associated with anastomotic leak (AL) and the outcomes related to this complication in elderly patients, using a large retrospective cohort from a high-volume colorectal surgery unit.

**Methods:**

A retrospective cohort study was conducted including patients aged ≥ 75 years who underwent colorectal cancer surgery between January 2015 and March 2019 at a tertiary centre. Univariable and multivariable logistic regression analyses were used to identify predictors of AL. Kaplan–Meier and Cox regression analyses assessed long-term survival.

**Results:**

A total of 416 patients (mean age 81.3 ± 4.7 years, 56% male) were included, with AL occurring in 12.26% (*n* = 51). Independent predictors of AL included preoperative hypoalbuminemia < 3 g/dL (OR 7.70, *p* = 0.009), mechanical anastomosis (OR 3.13, *p* = 0.033), and elevated CRP on postoperative day 3 (OR 1.14 per unit, *p* < 0.001). Intraoperative drain placement was protective (OR 0.34, *p* = 0.040). AL was associated with higher major complication rates, prolonged hospital stay, and increased 30-day and 5-year mortality (*p* < 0.0001). Discharge to rehabilitation centre or hospital-at-home discharge remained associated with AL (OR 4.57, *p* = 0.011). Independent predictors of mortality included emergency surgery, AL, ECOG ≥ 2, and Clinical Frailty Scale ≥ 5.

**Conclusions:**

These findings may support improved preoperative decision-making, patient counselling, and development of strategies to optimise outcomes in high-risk elderly patients.

## Introduction

The elderly population is progressively increasing in developed countries. This is mostly due to an improvement in healthcare systems with better control and management of comorbidities in the Western world. We are therefore facing an aging surgical population, with it being more common for elderly patients to attend colorectal units. At European level, 21% of the population was over 65 years in 2020 compared to 16% in 2001; 6% of the population were 80-year-olds in 2020 versus 3.4% in 2001 [1]. The World Health Organization (WHO) calculates that by 2050, a total of two billion people worldwide will be over 60 years representing 20% of the global population. Therefore, the WHO developed an action plan on aging and health in 2016, advocating for assessment of patient needs and to provide a personalised care plan with the goal of maintaining functional ability [2].

In colorectal cancer management the impact could be even larger, especially considering that colorectal cancer risk and incidence are greater in older patients. The burden of colorectal cancer is expected to increase in high-income countries in the coming years, and age is one of the risk factors related to this rising problem [3]. Understanding the management and special requirements of our elderly population may help improve our surgical outcomes and to develop strategies in our healthcare systems.

Anastomotic leakage (AL) is still one of the most feared complications in colorectal surgery. It is known to have an impact on postoperative morbidity and mortality rates [4, 5]. Different factors have been reported in the literature associated with AL risk: male gender, preoperative comorbidities, high American Society of Anesthesiologists (ASA) score, malnutrition, obesity, smoking, immunosuppression, neoadjuvant treatment, advanced tumour stage, low anastomoses, prolonged operative time, among other intraoperative conditions [6–8].

A better understanding of the risk factors for AL in older populations undergoing colorectal cancer resections can help to develop preventive strategies and improve postoperative care.

This study aimed to define these risks and explore long-term outcomes of AL in order to provide better assistance in colorectal cancer surgical management presented at older age.

## Methods

This was a retrospective cohort study from a prospectively maintained database from a colorectal surgery unit of a single tertiary centre. Elderly patients undergoing colorectal cancer surgery in our centre were consecutively included from January 2015 up to March 2019. Patients were followed up until 5 years after surgery. Younger patients were not included in this study for comparison as previously reported literature already assessed age groups as a factor for postoperative complications and/or leak. STROBE (Strengthening the Reporting of Observational studies in Epidemiology) guidelines were followed for the design and reporting of this study [9].

### Eligibility criteria

Patients younger than 75 years old at the time of the surgery were excluded. No maximum age limit was applied. Patients undergoing colorectal cancer surgery but without an enteric anastomosis were also not included. Procedures under any approach including open, laparoscopic and robotic surgery were included.

### Dataset

Data regarding preoperative management, patient comorbidities, as well as intraoperative factors and approach were collected. Postoperative 30-day complications including AL, mortality and length of stay were sought.

The variables included in this study for the preoperative period were demographic factors such as age and gender, but physical status assessments such as ASA classification, the Clinical Frailty Scale (CFS) [10], the Modified Frailty Index (mFI) [11] and ECOG performance status [12] were also collected and/or calculated from medical records retrospectively.

POSSUM physiological score, POSSUM mortality and operative severity scores [13] were also captured. For comorbidities smoking status, BMI, history of previous abdominal surgery, presence of a pre-existing stoma, ischaemic heart disease, congestive heart failure, cerebrovascular disease, diabetes mellitus, and immunosuppressive status were recorded. Preoperative albumin level when available, and oncological details as well as oncological stage, neoadjuvant and adjuvant therapy were collected. For intraoperative data urgency of the operation, surgical approach, primary operation performed, anastomosis creation and type (mechanical vs manual, intracorporeal vs extracorporeal, end to side vs side to side vs end to end), stoma formation, documented air leak or ICG perfusion test, operative duration, intraoperative complications using CLASSIC score [14] and whether intraoperative drains were used were included in the dataset. Postoperative outcomes recorded contained length of hospital stay, 30-day readmission, Clavien–Dindo complication grading [15], AL and its grading defined by Rahbari et al. [16], other 30-day complications, and postoperative inflammatory markers such as CRP on the third and fourth postoperative days, and PCT when available. Postoperative serum albumin was also included. Mortality was evaluated at 30 days, 90 days, 1 year, and 5 years. Cancer-related mortality was also captured.

A complete list of variables collected can be found in the study data collection form included in Appendix A as well as the scores used in the data collection.

### Study aims and outcome measures

The main aim was to assess different factors related to AL in colorectal cancer anastomosis in elderly patients over 75 years. The primary outcome measure was AL diagnosed in the postoperative period, whether this was managed conservatively or surgically. The definition and grading reported by the International Study Group of Rectal Cancer and Rahbari et al. were used [16].

In the unit, there is an existing protocol to proactively diagnose ALs early which includes obtaining CRP levels for all patients on the 3rd postoperative day. If CRP was over 140 mg/L, then 4th postoperative day CRP and PCT were evaluated. If 4th day CRP was over 125 and PCT was over 0.4 ng/mL, then a CT scan with intravenous contrast was performed adding rectal contrast if a rectal or low sigmoid anastomosis was present. Patients were reviewed daily, and clinical symptoms also triggered further investigations if AL was suspected.

As secondary outcome measures, postoperative morbidity based on Clavien–Dindo classification at 30 days and mortality at 30 days, 90 days, 1 year, and 5 years were taken into consideration.

### Statistical analysis

All datasets, analytical code, and results were electronically stored and archived at the Applied Statistics Service of the Autonomous University of Barcelona. Descriptive statistics were used to summarise the data. For quantitative variables data were reported using the number of observations, rates, mean, standard deviation and number of missing values; for qualitative variables data were presented as absolute and relative frequencies. Bivariate analyses were conducted to explore associations with the outcome of AL and mortality. For quantitative variables, the Shapiro–Wilk test was used to assess normality. Depending on the distribution, comparisons were made using either the parametric* t* test or the non-parametric Wilcoxon test. Associations between variables and AL were assessed using logistic regression models, with AL as the dependent variable. For each model,* p* values and odds ratios (OR) with 95% confidence intervals were reported for pairwise comparisons between categories. Statistical significance was set at a *p* value ≤ 0.05. Survival analysis was performed using Kaplan–Meier curves with log-rank tests to compare groups, and Cox proportional hazards models to identify independent predictors of mortality. Results were reported as hazard ratios (HR) with 95% confidence intervals, using a significance level of *p* value ≤ 0.05. Complete-case analysis was performed for the dataset rather than multiple imputation as the amount of missing data expected was generally low.

Statistical analysis was performed using SAS software version 9.4 (SAS Institute Inc., Cary, NC, USA). Multivariable logistic regression and survival analyses were performed using IBM SPSS Statistics version 20, and OR forest plots were generated using RStudio (R version 4.5.1) with the ggplot2 package on the Windows x86_64 platform.

### Ethical approval

Local ethics committee approval was obtained prior to the start of the study. As this was a retrospective review of patient data, the requirement of informed consent was waived. This study was conducted in accordance with the Declaration of Helsinki.

## Results

A total of 416 elderly patients who underwent colorectal cancer surgery were included in the study. The mean age was 81.3 ± 4.7 years, and 234 (56%) were male. Most patients (94.53%) had ASA II–III grade. The prevalence of comorbidities included history of ischaemic heart disease (10.82%) and congestive heart failure (10.58%). Mean preoperative albumin was 3.67 ± 0.606 g/dL and mean haemoglobin was 11.764 ± 1.958 g/dL. Regarding functional status and frailty, the mean mFI was 0.17 ± 0.137, and 10.1% had a Clinical Frailty Scale ≥ 5. Regarding oncologic staging and therapeutic strategies, the majority of patients presented with stage II or III colorectal cancer. Fifty-nine (14%) had a tumour located in the rectum. Forty patients (9.62%) received neoadjuvant therapy, which in 72% corresponded to combined chemotherapy and radiotherapy. In total, 105 patients received also adjuvant chemotherapy. Most of the procedures were elective (398; 95.6%) and performed via minimally invasive approaches such as laparoscopic (293; 70.43%) or robotic (49; 11.78%). AL occurred in 51 patients (12.26%), which, following the Rahbari et al. classification [16], were distributed as follows: grade A, 9 patients (2.16%); grade B, 14 patients (3.37%); and grade C, 28 patients (6.73%).

### Risk factors for AL

**Fig. 1:**
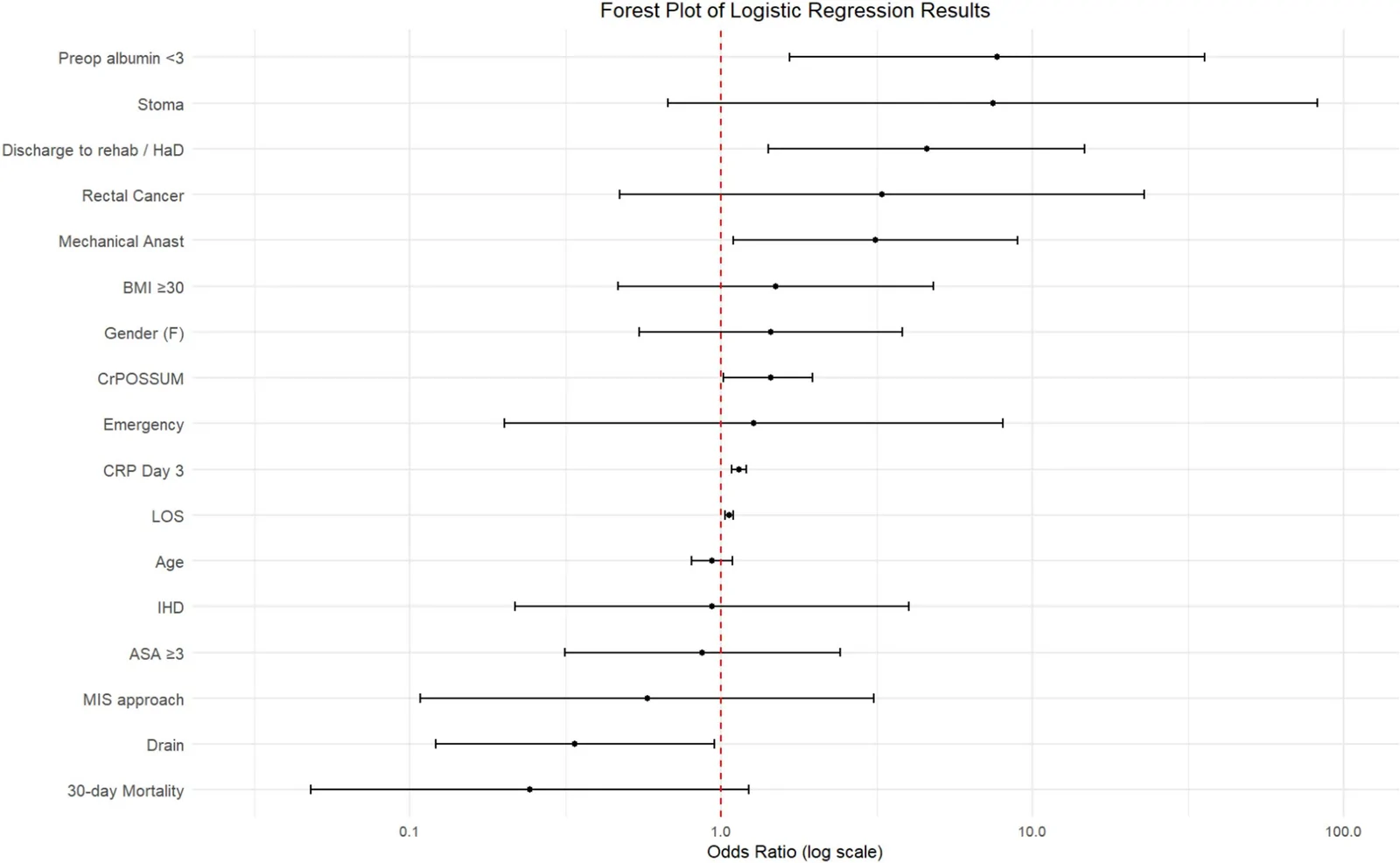
Forest plot of multivariable logistic regression of factors associated with AL

Risk factors associated with AL were evaluated. In the univariable analysis, higher ASA grade (*p* = 0.025), history of ischaemic heart disease (*p* = 0.010), and higher CR-POSSUM physiological score (*p* = 0.013) were identified as significantly associated with leak. Table 1 provides a complete list of the preoperative variables assessed. Frailty was a significant risk factor, with Clinical Frailty Scale ≥ 5 (*p* = 0.008) and ECOG ≥ 2 (*p* = 0.007) demonstrating higher leak rates. No differences were found when comparing oncological characteristics (Table 2) or intraoperative characteristics (Table 3) at univariable level. In the multivariable logistic regression, Table 4 and Fig. 1, preoperative albumin < 3 g/dL (OR 7.70, 95% CI 1.66–35.73, *p* = 0.009) and high CRP on postoperative day 3 (OR 1.14 per unit increase, *p* < 0.001) were identified as AL significant associated factors. Use of drains is not part of routine unit practice but they were used at the surgeons’ discretion when deemed required in 156 patients (37.5%). Intraoperative drain placement was significant a protective factor in the multivariable analysis (OR 0.34, *p* = 0.040).

**Table 1:** Preoperative characteristics, comorbidities, functional status and oncological stage of patients with AL

Variable	Levels	Leak	No leak	Missing values	*p* value
		*n* (%) or mean (± SD)	*n* (%) or mean (± SD)		
Age (years)		82.16 (± 4.56)	81.23 (± 4.73)	0	0.124
Gender	Male	33 (14.10%)	201 (85.90%)	0	0.196
	Female	18 (9.89%)	164 (90.11%)	0	
ASA	I	0 (0%)	6 (100%)	0	0.0255
	II	22 (11.64%)	167 (88.36%)	0	
	III	23 (11.27%)	181 (88.73%)	0	
	IV	6 (35.29%)	11 (64.71%)	0	
Smoker	Yes	5 (15.15%)	28 (84.85%)	0	0.1313
	Ex-smoker	20 (16.95%)	98 (83.05%)	0	
	No	26 (9.81%)	239 (90.19%)	0	
BMI		27.24 (± 4.32)	26.92 (± 3.95)	68	0.940
Previous abdominal surgery	Yes	20 (11.83%)	149 (88.17%)	0	0. 827
	No	31 (12.55%)	216 (87.45%)	0	
History of IHD	Yes	11 (24.44%)	34 (75.56%)	0	0.010
	No	40 (10.78%)	331 (89.22%)	0	
History of CHD	Yes	9 (20.45%)	35 (79.55%)	0	0.0848
	No	42 (11.29%)	330 (88.71%)	0	
Diabetes mellitus	Yes	13 (10.57%)	110 (89.43%)	0	0.496
	No	38 (12.97%)	225 (87.03%)	0	
Immunosuppressive treatment	Yes	1 (14.29%)	6 (85.71%)	0	0.869
	No	50 (12.22%)	359 (87.78%)	0	
Preoperative albumin (g/dL)		3.60 (± 0.58)	3.68 (± 0.61)	42	0.202
Preoperative haemoglobin		11.55 (± 2.26)	11.79 (± 1.91)	1	0.40
CR-POSSUM Physiological Score		12.39 (± 2.19)	11.50 (± 2.21)	1	0.0133
CR-POSSUM Mortality		10.53 (± 15.32)	8.85 (± 22.13)	1	0.0339
CR-POSSUM Operative Severity Score		7.35 (± 0.96)	7.48 (± 1.40)	1	0.832
Clinical Frailty Scale	1–3	31 (9.84%)	284 (90.16%)	0	0.008
	4	9 (15.25%)	50 (84.75%)	0	
	≥ 5	11 (26.19%)	31 (73.81%)	0	
Modified Frailty Index	Yes	0.21 (± 0.18)	0.16 (± 0.13)	0	0.152
Urgency	Elective	47 (11.81%)	351 (88.19%)	0	0.1973
	Urgent	4 (22.22%)	14 (77.78%)	0

**Table 2:** Oncological characteristics

Variable	Levels	Leak	No leak	Missing values	*p* value
		*n* (%) or mean (± SD)	*n* (%) or mean (± SD)		
Oncological T staging	TiS	3 (9.67%)	28 (90.32%)	0	0.7044
	T1	1 (3.57%)	27 (96.43%)	0	
	T2	12 (15.58%)	65 (84.42%)	0	
	T3	25 (12.38%)	177 (87.62%)	0	
	T4	10 (12.82%)	68 (87.18%)	0	
Oncological N staging	N0	32 (12.40%)	226 (87.60%)	0	0.6915
	N1	12 (10.62%)	101 (89.38%)	0	
	N2	7 (15.56%)	38 (84.44%)	0	
Oncological M staging	M0	48 (12.21%)	345 (87.79%)	0	0.9061
	M1	3 (13.04%)	20 (13.04%)	0	
Neoadjuvant treatment	Yes	4 (10%)	36 (90%)	0	0.647
	No	47 (12.50%)	329 (87.50%)	0	
Adjuvant treatment	Yes	9 (8.41%)	98 (91.59%)	0	0.1631
	No	42 (13.59%)	267 (86.41%)	0	
ECOG ≥ 2	0–1	41 (10.8%)	339 (89.2%)	0	0.007
	≥ 2	10 (27.8%)	26 (72.2%)	0	
Cancer diagnosis location	Colon	45 (26%)	312 (87.4%)	0	0.597
	Rectum	6 (10.2%)	53 (89.8%)	0

**Table 3:** Intraoperative characteristics

Variable	Levels	Leak	No leak	Missing values	*p* value
		*n* (%) or mean (± SD)	*n* (%) or mean (± SD)		
Minimally invasive approach	Laparoscopic	32 (10.92%)	261 (89.08%)	0	0.3359
	Laparoscopic converted to open	2 (10.53%)	17 (89.47%)	0	
	Open	10 (18.87%)	43 (81.13%)	0	
	Robotic	6 (12.24%)	43 (87.76%)	0	
	Robotic converted to open	1 (50%)	1 (50%)	0	
Anastomosis technique	Manual	17 (10.56%)	144 (89.44%)	0	0.4018
	Mechanical	34 (13.33%)	221 (86.67%)	0	
Site of anastomosis formation	Extracorporeal	25 (11.85%)	186 (88.15%)	0	0.7953
	Intracorporeal	26 (12.68%)	25 (11.85%)	0	
Anastomosis configuration	End to end	21 (13.91%)	130 (86.09%)	0	0.6355
	End to side	3 (13.04%)	20 (86.96%)	0	
	Side to end	3 (20%)	12 (80%)	0	
	Side to side	23 (10.65%)	193 (89.35%)	0	
Stoma formation	No	47 (12.5%)	329 (87.5%)	0	0.4354
	Yes	4 (10%)	36 (90%)		
Intraoperative complications	No	31 (10.30%)	270 (89.70%)	0	0.0510
	Yes	20 (17.39%)	95 (82.61%)		
Duration of procedure (min)		225.69 (± 70.66)	215.22 (± 62.65)	1	0.3026
Intraoperative drain	No	26 (10.08%)	232 (89.92%)	0	0.1564
	Yes	23 (14.74%)	133 (85.26%)

**Table 4:** Multivariable logistic regression analysis for factors related to AL

Variable	OR	Lower 95% CI	Upper 95% CI	*p* value
CrPOSSUM	1.441	1.015	1.961	0.40
Age	0.936	0.805	1.089	0.392
Gender (female)	1.443	0.545	3.823	0.460
ASA ≥ 3	0.871	0.314	2.413	0.790
IHD	0.934	0.218	3.999	0.927
BMI ≥ 30	1.495	0.465	4.801	0.500
Preop albumin < 3	7.701	1.660	35.728	0.009
Rectal Q	3.287	0.473	22.844	0.229
Emergency	1.270	0.201	8.010	0.799
MIS approach	0.579	0.108	3.089	0.522
Mechanical anastomosis	3.129	1.094	8.947	0.033
Stoma	7.445	0.673	82.352	0.102
Drain	0.339	0.121	0.953	0.040
CRP day 3	1.138	1.078	1.201	< 0.0001
LOS	1.061	1.031	1.091	< 0.0001
Discharge to rehabilitation/hospital-at-home	4.565	1.421	14.667	0.011
30-day mortality	0.243	0.048	1.225	0.087

Although CFS was significant in univariable analysis, it did not retain independent significance in the multivariable models. This may be due to collinearity with other patient risk factors such as age and physiological status. Additionally, exclusion of CFS improved model sensitivity, maximising detection of ALs. Therefore, CFS was excluded from the final model.

In this study, mechanical anastomosis was not significant in univariable analysis but became significant in one of the multivariable models (OR 3.13, 95% CI 1.09–8.95, *p* = 0.033). This suggests that adjustment for confounding variables revealed an independent association. However, its lack of significance in alternative multivariable models indicates that the finding is sensitive to model specification and may be influenced by collinearity or sample variability. Therefore, results for this variable should be interpreted with caution.

### Clinical impact of AL

**Fig. 2:**
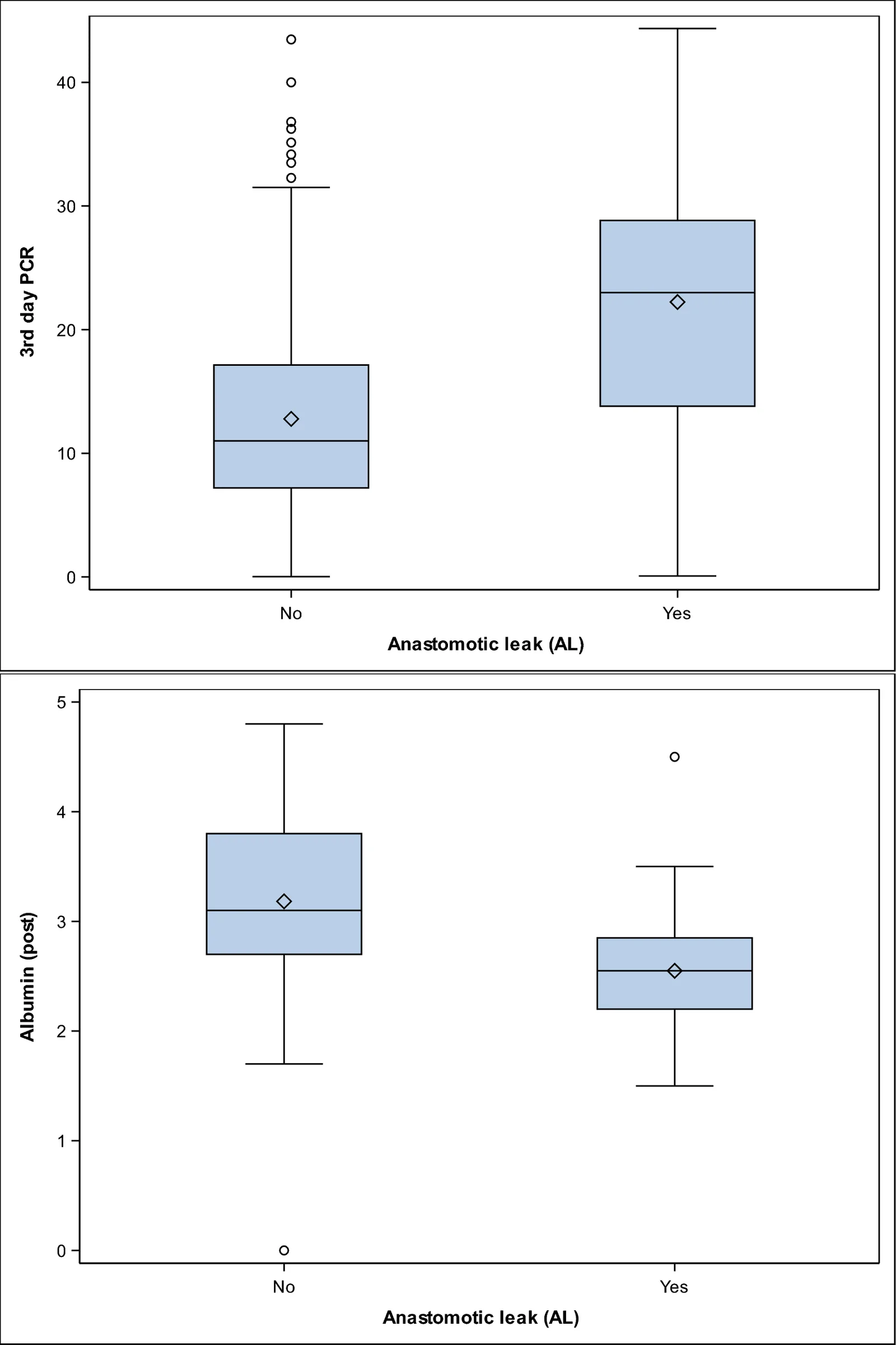
Box plot for analytic values statistically significant in the univariable analysis

AL was associated with significantly higher rates of major complications Clavien–Dindo ≥ 3 (*p* < 0.0001), and an increased 30-day and 5-year mortality (*p* < 0.0001). In the postoperative period hypoalbuminemia seems to be significantly associated with leak 2.55 ± 0.55 vs 3.18 ± 0.72 (Table 5, Fig. 2). Some of the important factors identified to correlate with AL in the univariable and multivariable analysis were length of hospital stay (OR 1.06 per day, *p* < 0.001), and discharge to a rehabilitation/recovery centre or “hospital-at-home” (OR 4.57, *p* = 0.011).

**Table 5:** Postoperative outcomes

Variable	Levels	Leak	No leak	Missing values	*p* value
		*n* (%) or mean (± SD)	*n* (%) or mean (± SD)		
30-day major complications—CD ≥ 3	No	16 (4.46%)	343 (95.54%)	0	< 0.0001
	Yes	35 (61.40%)	22 (38.60%)	0	
3rd POD CRP		22.24 (± 10.69)	12.78 (± 8.13)	118	< 0.0001
Postoperative albumin (g/dL)		2.55 (± 0.55)	3.18 (± 0.72)	44	< 0.0001
Readmission	No	47 (12.40%)	332 (87.60%)	0	0.7785
	Yes	4 (10.81%)	33 (89.19%)		
LOS (days)		26.80 (± 16.56)	9.15 (± 11.91)	0	< 0.0001
30-day mortality	No	42 (10.50%)	358 (89.50%)	0	< 0.0001
	Yes	9 (56.25%)	7 (43.75%)	0	
Discharge to rehab centre or home hospitalisation	No	32 (8.8%)	332 (91.2%)	0	< 0.0001
	Yes	19 (36.5%)	33 (63.5%)		
5-year mortality	No	18 (6.95%)	241 (93.05%)	9	< 0.0001
	Yes	32 (21.62%)	116 (78.38%)		
5-year cancer-related mortality	No	40 (9.6%)	312 (75%)	4	0.237
	Yes	11 (2.6%)	49 (11.8%)

### Long-term mortality analysis

**Fig. 3:**
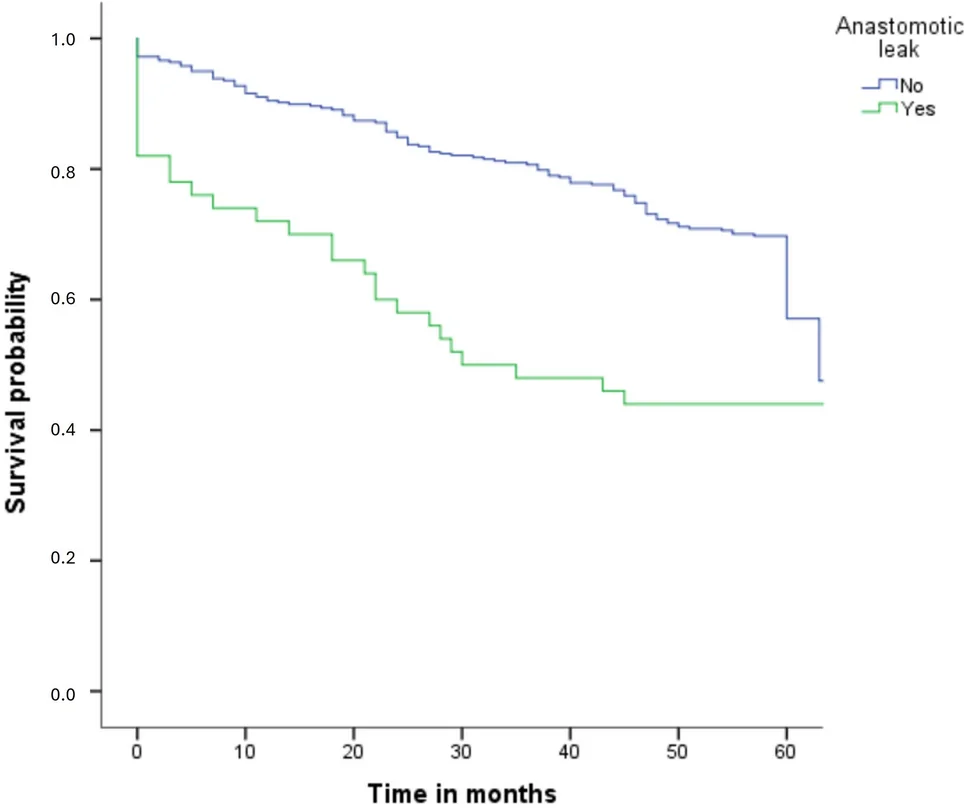
Kaplan–Meier overall survival, leak vs no leak (*p* < 0.0001)

The Kaplan–Meier curve (Fig. 3) illustrates a statistically significant difference in survival between patients who experienced an AL and those who did not (log-rank test: χ^2^ = 14.493, df = 1, *p* < 0.001) in a follow-up of 5 years.

No significant association was found between mortality related to disseminated/recurrent oncological disease and AL (*p* = 0.237) in the univariable analysis or multivariable.

Survival analysis using Cox regression demonstrated that emergency surgery (HR 0.268, *p* < 0.001), worse ECOG performance status (HR 1.665, *p* = 0.001), and higher Clinical Frailty Scale scores (HR 1.215, *p* = 0.02) were independently associated with increased mortality (Table 6). Oncological stage was also significant overall (*p* = 0.02). AL showed a trend towards increased mortality (HR 1.543, *p* = 0.05).

A total of nine patients were lost in the 5-year long-term follow-up.

**Table 6:** Multivariable Cox regression results

Variable	HR (exp(*B*))	*p* value
Age	1.032	0.18
IHD	0.876	0.60
Physiology score	1.000	0.99
Oncological stage (overall)	–	0.02
Diagnosis	1.438	0.21
Urgency	0.268	< 0.001
Preop albumin (< 3 g/dL)	1.193	0.31
Preop albumin (≥ 3 g/dL)	0.913	0.51
ECOG	1.665	< 0.001
AL	1.543	0.05
Clinical Frailty Scale	1.215	0.02
Modified Frailty Index	1.016	0.21

## Discussion

This study evaluated risk factors, clinical impact and long-term outcomes of AL following colorectal cancer surgery in older patients. The overall leak rate in this cohort was 12.26%, with most leaks classified as grade C (*N* = 28) and therefore undergoing surgical management. The rates are in accordance with those reported in the literature, typically between 1% and 19% depending on anastomosis location (colon vs rectum), patient comorbidities and surgical complexity [17]. For older patients over 75 years old some of the literature reports rates around 8–10% [18, 19].

Among the factors we found significantly related to AL, there were patient-related comorbidities including ASA grade, previous history of ischaemic heart disease, or postoperative hypoalbuminemia. High CR-POSSUM Physiological Score was also related to higher risk for leak. Some of these factors have already been pointed out by some authors to be important in older age groups, highlighting nutritional status, ASA grade or adherence to ERAS protocols in perioperative management [20–22].

Nutritional status, reflected by hypoalbuminemia, was an independent predictor of leak, aligning with evidence that malnutrition impairs healing. This supports prehabilitation strategies focusing on nutritional optimisation before surgery to reduce risk. We did not measure sarcopenia in this study, but this is a common factor that can be accentuated in both older age and cancer. It has already been described as an independent factor for complications and leak [23, 24]. A decreased level of serum albumin in the preoperative or early postoperative days can be an indirect reflection of sarcopenia [25].

In our cohort we did not found differences in the intraoperative factors related to open vs laparoscopic vs robotic surgical approach; however, in the literature, MIS has been proven to be safe and have better short-term outcomes [26–28]. Mechanical anastomosis was found to have a higher risk for leak in the multivariable analysis, but this needs to be interpreted with caution as it did not retain significance in alternative multivariable models and therefore its result may be influenced by collinearity or sample variability.

Regarding oncological characteristics, tumour stage and neoadjuvant or adjuvant treatment were similar between AL and no-leak groups. Interestingly, the only factor that was higher in the leak group and later related to a higher risk for mortality was an ECOG higher than 2, which represents a worse functional status. AL did not seem to be associated with increased cancer-related mortality or oncological recurrence for this group. This contrasts with the available literature which has suggested that AL is associated with local recurrence and reduced long-term survival [29]. However all the published meta-analyses on the topic combine both a young and an elderly population.

Few studies exist in current literature studying the impact of frailty in older populations undergoing colorectal cancer and their outcomes. Frailty was a significant determinant, with a Clinical Frailty Scale score ≥ 5 indicating clinical frailty and associated with higher AL risk and mortality. No significant differences were seen with the Modified Frailty Index. These results align with the frailty scoring meta-analysis recently published by Ding et al. [30]. Previous literature highlights the elderly’s vulnerability due to poor physiological reserve, other comorbidities, and functional status, as a combined risk for poor outcomes in cases where an AL happens [31, 32]. Therefore, AL may represent both an independent adverse event and a factor of underlying patient vulnerability. These findings support the need for preoperative frailty assessment as part of surgical decision-making and as part of the information given to the patient and relatives at time of consent or when dealing with expectations after a complication. Another interesting finding associated with leak is an increased risk to require further hospitalization in a rehabilitation centre or at home due to IV antibiotic requirement. The length of stay was also longer in the patients with leak. This result may be of use when preparing strategies for efficiency in the department and better planning given the risk.

Some strategies that have been part of the standard protocol of the colorectal unit at Vall d’Hebron Hospital for many years are a blood test on the third postoperative day including the CRP and further strategies in the algorithm explained above in the “Methods” section. A high CRP is an independent factor for leak in our study in the univariable and multivariable analyses. This is a cheap biomarker that can help in the early tracking and detection of AL after surgery. In a similar way, intraoperative drain was found to be a protective factor for leak. However, previous larger cohort studies in colorectal surgery have reported drains not to prevent postoperative collections [33] and are not part of the ERAS recommendations from which octogenarian patients could benefit [34, 35].

Though multivariable Cox regression showed no difference association between survival and age for the population over 75, functionality and frailty remained important, with ECOG over 2 and higher CFS emerging as independent predictive factors of mortality in this cohort.

The relationship between AL and long-term survival is likely multifactorial. Frailty, impaired functional status, and malnutrition increase the risk of AL while independently predisposing patients to poorer survival. At the same time, AL may accelerate the decline of the patient functional status and therefore increase length of hospitalisation, loss of independence, and reduced physiological reserve, effects that may be particularly pronounced in elderly patients. Therefore, leakage should be considered both a postoperative complication and as a marker of patient vulnerability rather than interpreted as an isolated cause of reduced long-term survival.

### Limitations

This study is not without limitations. Some of them are inherent to its retrospective design and single-centre setting. The aim of the study was to further explore the outcomes of an elderly cohort, and as multiple previous literature exists comparing elderly with a younger population, this was not included in our project. Also, overall mortality and cancer-related mortality were the events captured, but future studies would benefit from further exploring detailed mechanisms and factors in the increased non-cancer mortality. Recurrence and disease-free survival were not evaluated as endpoints for this study; however, future studies should explore these outcomes too.

The strengths of this study include a large, well-characterised elderly cohort with comprehensive long-term follow-up in a high-volume certified colorectal unit.

## Conclusions

These findings highlight the importance of shared decision-making, incorporating frailty, nutritional status, and prehabilitation in the colorectal cancer management plans in the elderly, especially in the consent discussions and preparation before surgery. Moreover, these results support the necessity of preoperative and postoperative interventions aiming to reduce AL. Some suggested strategies would include better optimisation before surgery (frailty assessment, nutrition improvement and prehabilitation) and early postoperative detection of the AL to reduce the long-term impact.

The results from this study may help to design future trials and prospective studies on specific strategies that may benefit an older population where a higher risk of complications is expected. Further studies regarding the true impact of frailty could be beneficial for better understanding of outcomes for elderly undergoing colorectal cancer surgery and therefore helping in the guidance of their management.

## Data Availability

No datasets were generated or analysed during the current study.
